# Increased expression of CD36 and CD163 in clear cell renal cell carcinoma suggests an association between lipid transport and an “M2-like” macrophage phenotype

**DOI:** 10.3389/fimmu.2026.1773666

**Published:** 2026-03-02

**Authors:** Yosra Bouraoui, Rahma Said, Christina Bruss, Agnieszka Martowicz, Kilian Wagner, Florian Weber, Simon U. Engelmann, Sebastian Kaelble, Marcus Höring, Gerhard Liebisch, Roman Mayr, Peter J. Siska

**Affiliations:** 1 Laboratory of Functional Physiology and Valorization of Bioresources, Higher Institute of Biotechnology of Beja, University of Jendouba, Beja, Tunisia; 2 Higher Institute of Biotechnology of Beja, University of Jendouba, Beja, Tunisia; 3 Department of Internal Medicine III, Hematology and Medical Oncology, University Hospital Regensburg, Regensburg, Germany; 4 Institute of Functional Genomics, University of Regensburg, Regensburg, Germany; 5 Department of Internal Medicine V, Haematology & Oncology, Comprehensive Cancer Center Innsbruck (CCCI) and Tyrolean Cancer Research Institute (TKFI), Medical University of Innsbruck, Innsbruck, Austria; 6 Institute for Pathology, University of Regensburg, Regensburg, Germany; 7 Department of Urology, Caritas St. Josef Medical Centre, University of Regensburg, Regensburg, Germany; 8 Institute of Clinical Chemistry and Laboratory Medicine, University Hospital Regensburg, Regensburg, Germany; 9 Urology, Faculty of Medicine, University of Augsburg, Augsburg, Germany

**Keywords:** CD163, CD36, kidney cancer, lipids, macrophages

## Abstract

**Background:**

Clear cell renal cell carcinoma (ccRCC) is marked by intracellular lipid accumulation and dysregulated lipid metabolism, features that contribute to tumor progression and possibly immune suppression. Tumor-associated macrophages (TAMs) in ccRCC are abundant and phenotypically diverse, with CD68 marking general macrophage presence and CD163 indicating alternatively activated, immunosuppressive (M2-like) subsets. The fatty acid transporter CD36 and the metabolic regulator CD147 can be found in tumors and may influence macrophage polarization, but their associations with TAM phenotypes and tumor lipid content in ccRCC remain unclear.

**Methods:**

Samples from 23 ccRCC patients were analyzed. Oil Red O staining quantified lipid accumulation. Immunohistochemistry for CD36, CD147, CD68, CD163, and CD3 was evaluated using both automated area-based quantification and semi-quantitative observer scoring. Flow cytometry of freshly resected tumors was used to assess the expression of CD36, CD147 and the infiltration with macrophages and CD8 T cells. Lipidomic analyses were performed from ccRCC tissues (n=5). RNA expression data from TCGA (n=533) were used for validation. Single-cell RNA sequencing data from two published datasets were analyzed to characterize cell-type-specific expression of lipid metabolism and macrophage markers.

**Results:**

ccRCC tumors showed an increased lipid accumulation and the expression of CD36 was detected on both CD45-negative cells and macrophages. Macrophages expressed CD163, suggesting a M2-like phenotype and CD163 expression was higher in larger ccRCC tumors. CD163 on macrophages positively correlated with CD36 on CD45-negative cells from ccRCC tumors, while CD8 T cells showed a trend to lower numbers in tumors with high CD36 expression on CD45-negative cells. CD147 was broadly expressed on CD45-negative and CD45-positive cells and positively correlated with CD36 on CD45-negative cells as well as with CD163 on macrophages. Tumors accumulated triacylglycerols, which correlated with CD163. Single-cell RNA-seq revealed CD163 expression across all TAM subsets and a compartmentalized distribution of lipid metabolism genes.

**Conclusions:**

Lipid metabolic markers CD36 and CD147 are expressed in ccRCC tumors and correlations with immune cell subsets suggest their role in suppressing anti-tumor immunity. These findings suggest the existence of a metabolic-immune axis in ccRCC and provide a rationale for targeting TAM metabolism to enhance immunotherapeutic efficacy.

## Background

Clear cell renal cell carcinoma (ccRCC) is the most prevalent histological subtype of renal cancer, characterized by the accumulation of intracellular lipids and glycogen. This distinctive metabolic phenotype reflects alterations in lipid metabolism, now recognized as a hallmark of ccRCC biology (1–3). To sustain their rapid growth and survival, ccRCC cells rely on increased uptake of exogenous lipids and upregulation of *de novo* lipogenesis and cholesterol biosynthesis. These metabolic adaptations support membrane synthesis, energy storage, and signaling, contributing to aggressive tumor behavior and therapy resistance.

Among lipid uptake regulators, CD36 plays a critical role in fatty acid transport and has been implicated in macrophage lipid accumulation and immunosuppressive reprogramming (4–6). Su et al. reported the key role of CD36 on tumor-associated macrophages for lipid accumulation and tumor promotion in a murine model (7). Recently, Xu et al. showed that CD36 represses the type-I interferon signaling in tumor associated macrophages (8). Interestingly, increased CD36 expression might be a consequence, rather than a driver of an immunosuppressive TAM phenotype, although this pathway is not yet completely understood (6). CD36 expression was found increased in ccRCC and correlated with systemic lipid metabolic state (9). However, CD36 can also be involved in renal pathologies such as diabetic kidney disease (10) or metabolic syndrome-associated renal injury (11). Therefore, CD36 represents a key structure for studying lipid metabolic pathways and a possible target for treatment of malignant and non-malignant diseases.

CD147 (also known as basigin or EMMPRIN), a transmembrane glycoprotein involved in extracellular matrix remodeling and metabolic regulation, has been associated with tumor progression and immune evasion as well as immune dysfunction (12, 13). Furthermore, CD147 is a regulator of fatty acid metabolism (14). As a multi-functional metabolic facilitator at the plasma membrane, it interacts with caveolin-1, MCTs, and potentially with lipid raft–associated molecules, which are critical in lipid trafficking (15). While studied in lung adenocarcinoma (16), hepatocellular carcinoma (14) and colorectal cancer (17) where it can regulate tumor metabolic pathways, a possible metabolic role of CD147 has not been studied in RCC so far.

Several immune cell populations can be found in the ccRCC tumor microenvironment (TME) (18). Notably, T cells have been studied for their role in RCC, with evidence suggesting metabolic impairments affecting their function (19). ccRCC tumor-associated macrophages (TAM) can differentiate into pro- (M1-like) and anti-inflammatory (M2-like) phenotypes and might represent a promising therapeutic target (20). TAMs are abundant in RCC and exhibit considerable phenotypic plasticity. Their functional polarization is increasingly recognized to be influenced by local metabolic cues, especially lipid availability (21). However, the extent to which lipid metabolism in RCC directly contributes to TAM polarization remains insufficiently explored.

CD163, a scavenger receptor commonly used as a marker of alternatively activated (M2-like), tumor-promoting macrophages, is also enriched in TAM populations in tumors (22). Its expression may reflect an immunosuppressive TAM phenotype shaped by lipid-rich environments, although its relationship with CD36- and CD147-mediated metabolic pathways remains to be elucidated.

In this study, we investigate the expression of CD36, CD147, and CD163 in ccRCC tumor tissues to study how lipid metabolic cues may influence TAM differentiation and function. We combine analyses of protein expression, lipid accumulation, flow cytometry and RNA-sequencing data from public datasets to dissect potential links between tumor lipid metabolism and macrophage polarization. Our findings aim to uncover novel metabolic-immune crosstalk mechanisms and provide candidate targets for reprogramming TAMs to enhance immunotherapeutic strategies in ccRCC.

## Methods

### Patient cohort and clinical data

Human kidney tissue samples were collected from patients (n=23) with suspected clear cell renalcell carcinoma (ccRCC) at the Department of Urology, University Hospital Regensburg. The diagnosiswas later confirmed at the Department of Pathology, University Hospital Regensburg. Normal tissue was obtained from unaffected regions of the same kidney. Tumor tissue was identified and marked for analysis by an experienced pathologist, subsequently the % of malignant tissue in the tumor sample was determined (**Supplementary Table S1**). For the analyses of freshly resected samples (n=11), three anatomically definedregions—normal kidney, tumor periphery, and tumor center—were selected based onpathological assessment. Ethical approval was obtained from the local ethics committee (University of Regensburg vote number: 16-355-101) and conducted in accordance with the Declaration of Helsinki. All patients provided informed consent. Clinical details are summarized in **Supplementary Table S1**.

### Immunohistochemistry and quantification

Frozen tissue sections (5 μm thick) were fixed in 4% paraformaldehyde for 20 minutes. Antigen retrieval was performed using 10 mmol/L sodium citrate buffer (pH 6.0, Zytomed) heated to 92–98 °C for 20 minutes. After PBS washing, endogenous peroxidase activity was blocked using Dako REAL peroxidase blocking solution for 5 minutes at room temperature.

Slides were incubated overnight at 4 °C with the following primary antibodies: Mouse anti-CD163 (1:100, Invitrogen), Mouse anti-CD36 (1:200, StemCell), Mouse anti-CD68 (1:200, Dako), Rabbit anti-CD147 (1:250, GeneTex). After washing, secondary antibody incubation was performed using a universal HRP-conjugated polymer (Histofine Simple Stain Max PO, anti-rabbit and anti-mouse) for 30 minutes in a dark room. Signal detection was carried out with DAB substrate (Vector, SK-4100) for 2 minutes, followed by hematoxylin counterstaining.

Representative images were acquired using an ECHO Rebel light microscope. For each tissue sample, three independent 4 µm sections were analyzed. In each section, six random fields were examined at 40x magnification using Fiji/ImageJ software. Staining intensity was quantified as optical density per unit area, calculated from the mean gray value of each region of interest after grayscale conversion and background subtraction. In addition, a trained observer, blinded to sample identity and experimental grouping, assigned semi-quantitative scores ranging from 1 (lowest) to 9 (highest) based on both staining intensity and the proportion of positive cells. These two methods were performed concurrently and used to validate each other. Correlation analyses were primarily based on observer-derived scores using Pearson´s correlation coefficients with two-tailed p-values, and plots were generated using GraphPad Prism.

### Lipidomics

Tissue homogenates representing a wet weight of 2 mg were extracted according to the method of Bligh and Dyer (23) in the presence of not naturally occurring lipid species as internal standards.

Lipid species were quantified by direct flow injection analysis (FIA) using a triple quadrupole mass spectrometer (FIA-MS/MS) and a high-resolution hybrid quadrupole-Orbitrap mass spectrometer (FIA-FTMS). FIA-MS/MS was performed in positive ion mode using the analytical setup and strategy described previously (24, 25). A fragment ion of *m/z* 184 was used for lysophosphatidylcholines (LPC) (26). The following neutral losses were applied: Phosphatidylethanolamine (PE) and lysophosphatidylethanolamine (LPE) 141, phosphatidylserine (PS) 185, phosphatidylglycerol (PG) 189 and phosphatidylinositol (PI) 277. Sphingosine based ceramides (Cer) and hexosylceramides (Hex-Cer) were analyzed using a fragment ion of *m/z* 264 (27). Glycerophospholipid species annotation was based on the assumption of even numbered carbon chains only.

A detailed description of the FIA-FTMS method was published recently (28, 29). Triglycerides (TG), diglycerides (DG) and cholesterol esters (CE) were recorded in positive ion mode as [M+NH_4_]^+^ at a target resolution of 140,000 (at 200 *m/z*). CE species were corrected for their species-specific response (30). Phosphatidylcholines (PC) and sphingomyelins (SM) were analyzed in negative ion mode as [M+HCOO]^-^ at the same resolution setting. Multiplexed acquisition (MSX) was applied for the determination of free cholesterol (FC) and the respective internal standard (FC[D7]) (30).

### Oil Red O staining

Cryopreserved tumor and matched normal kidney tissue blocks were sectioned using a cryostat and stored at –80°C. Before staining, slides were air-dried at room temperature and washed with PBS. Lipid staining was performed using Oil Red O (ScienCell, #0843-SC) according to the manufacturer’s instructions. Nuclear counterstaining was performed with Hemalaun (Merck, #1.09249.0500), and slides were mounted with Faramount Aqueous Mounting Medium (Dako, #S3025). Representative images were acquired using an ECHO Rebel microscope.

For quantification, slides were thawed for 2.5 hours at room temperature, fixed for 15 minutes, and rinsed with distilled water. Oil Red O working solution (600 µl stock + 400 µl distilled water, filtered through 0.2 µm) was applied for 15 minutes. Slides were washed thoroughly with tap water, counterstained with hematoxylin for 3 minutes, and mounted. Lipid droplets appeared red, and nuclei blue.

### Multiplex immunofluorescence imaging

Cryosections from three RCC patients (also included in the IHC cohort) were mounted on coated slides and fixed with 4% paraformaldehyde. Multiplex immunofluorescence staining was performed using the MACSima™ Imaging Platform (Miltenyi Biotec), an automated system for cyclic staining and imaging. Reagents and antibodies used included FcR Blocking Reagent (Cat# 130-059-901), CD36 PE (Cat# 130-110-877), CD147 APC (Cat# 130-124-295), CD8a PE (Cat# 130-117-201), CD45 PE (Cat# 130-113-118), CD68 PE (Cat# 130-128-345), CD163 PE (Cat# 130-127-908), and pan-Cytokeratin APC (Cat# 130-123-091), all from Miltenyi Biotec. DAPI (D9542) was purchased from Sigma Aldrich. All steps, including staining and imaging, were performed according to the manufacturer’s protocol.

### Tissue processing and flow cytometry

Tissues were processed as described previously (19). Briefly, samples were minced and enzymatically digested using Collagenase IV and DNase I to generate single-cell suspensions. After filtration through a 70 µm mesh, cells were washed, counted, and stained with fluorochrome-conjugated antibodies for surface marker analysis by flow cytometry. The following antibodies were used: CD3 V450 (BD, Cat# 560365), CD45 PerCP (BioLegend, Cat# 304026), CD8 FITC (BioLegend, Cat# 344704), CD68 PE-Cy7 (BioLegend, Cat# 333816), CD163 BUV496 (BD, Cat# 752878), CD36 PE (Miltenyi Biotec, Cat# 130-110-877), CD147 APC (BioLegend, Cat# 306214). Flow cytometric acquisition was performed on a BD LSRFortessa™ instrument, and data were analyzed using FlowJo v10 (BD Biosciences).

### RNA-seq data

Bulk RNA sequencing data of 533 ccRCC patients were retrieved from The Cancer Genome Atlas (TCGA) (KIRC dataset) (31, 32) using the UCSC Xena platform (33). Gene expression values are reported as log2(fpkm-uq + 1).

First cohort of single-cell RNA sequencing data from 12 RCC patients was obtained from previouslypublished work by Li R. et al. (34), available as anAnnData (.h5ad) object, Dataset (35). The dataset was analyzed using Scanpy v1.9.1. Quality control filtering excluded cells with fewer than 200 detected genes, fewer than 2000 total counts, or more than 30% mitochondrial transcript content. To correct for batch effects and enable downstream analysis, cell transcriptomes were embedded into a low-dimensional latent space using scVI (36, 37), treating each patient sample as a distinct batch. The model was trained on the 2000 most highly variable genes, identified using Scanpy’s pp.highly_variable_genes function with the parameters flavor=“seurat” and batch_key=“orig.ident”. A neighborhood graph and UMAP embedding were generated from the resulting scVI latent space. Cell type annotations were adopted from the original publication (34). For gene set enrichment analysis, over-representation analysis (ORA) of hallmark gene sets was performed using the decoupler-py package v.2.1.1 (38). To quantify gene co-expression, double-positive cells were defined as those expressing ≥1 UMI for both genes of interest, and fraction of positive cells were calculated based on the number of cells with ≥1 UMI per gene. This dataset includes non-malignant tissues from ccRCC patients (**Supplementary Figure S2F**), the focus on specific sub-populations is described in the corresponding figure legend.

Second cohort of single-cell transcriptomic data from human ccRCC samples were analyzed using a publicly available dataset published by Bi et al. (18). Data visualization and exploration were conducted via the Single Cell Portal (39). Cell types, including immune subpopulations, were annotated as defined by the original authors. Marker expression was examined across these distinct immune compartments to investigate cell type–specific expression profiles relevant to the tumor microenvironment. The cohort consisted of eight ccRCC patients with five patients having received immune checkpoint blockade (ICB), including four who had also been treated with tyrosine kinase inhibitors (TKIs). The remaining three patients were treatment-naïve (18).

### Statistical analysis

Data are expressed as mean and individual values for microscopy data and as violin plots with median and quartiles for the transcriptomic data. Statistical comparisons were performed using one-way ANOVA with *post hoc* tests, and Student’s t-test, as appropriate, using GraphPad Prism. Correlation analyses between markers were assessed by Pearson´s correlation with two-tailed p-values. Significance levels were set as * p<0.05, ** p<0.01, *** p<0.001,**** p<0.0001.

## Results

### Increased CD36 expression and prominent lipid accumulation in ccRCC

**Figure 1:**
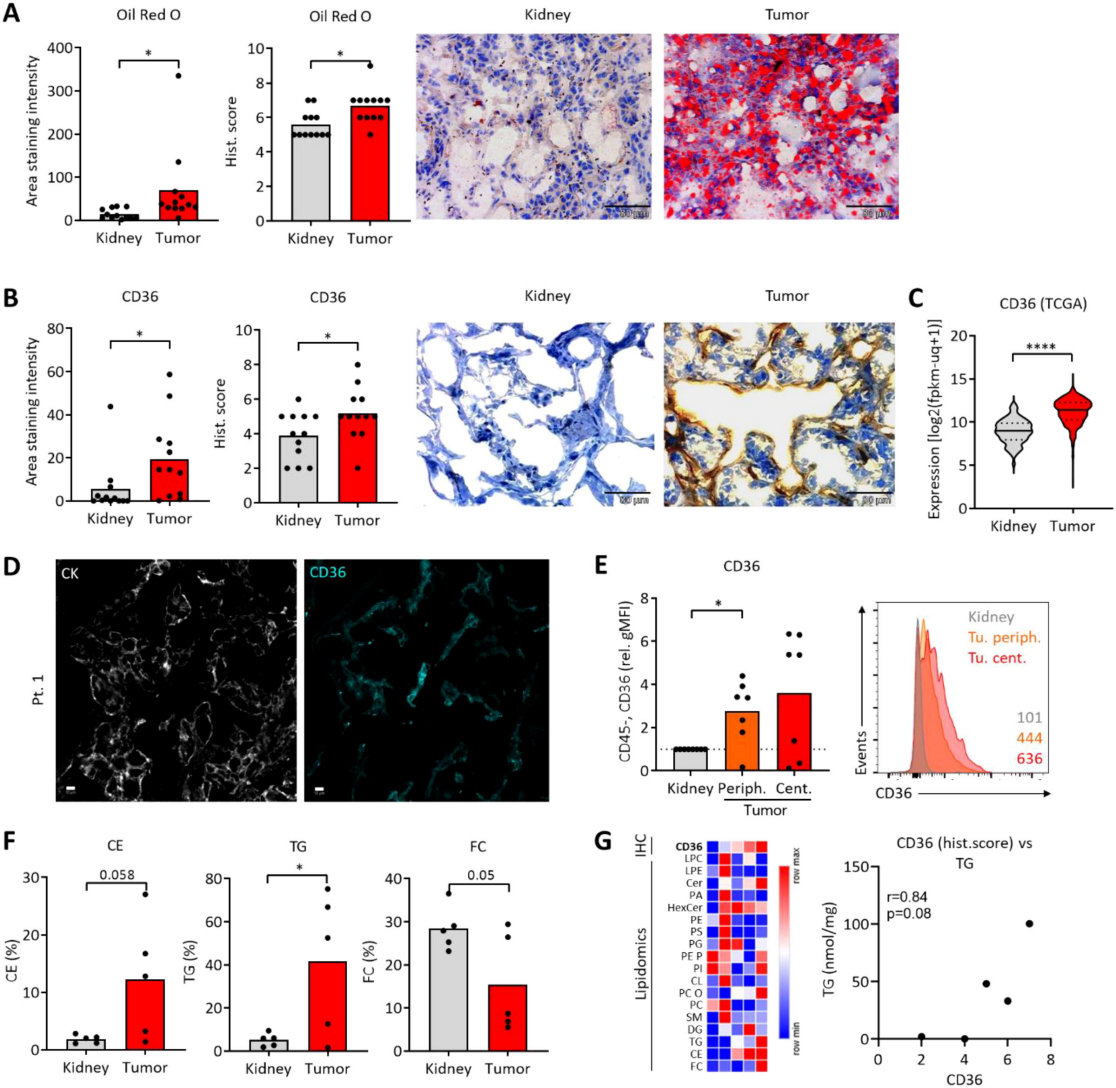
Increased CD36 expression and prominent lipid accumulation in ccRCC samples. Immunohistochemistry data was analyzed using two complementary approaches: (1) Quantitative image analysis providing the Area staining intensity, and (2) Observer-based scoring, providing the Histological score. For more details refer to Material and Methods section. **(A)** Oil Red O was used to visualize lipid accumulation in ccRCC tumors and adjacent kidney tissues. **(B)** CD36 was assessed using immunohistochemistry. Representative images in **(A, B)** are not matched from the same patient due to insufficient tissue pairing during sample collection. **(C)** Expression of CD36 was assessed using the available TCGA data (KIRC dataset). Student’s t-test, *p<0.05, ****p<0.0001. **(D)** Multiplex immunofluorescence imaging of ccRCC tumor tissues (representative of three patients) depicting CK (cytokeratin) and CD36 expression. White bar represents 10 μm. **(E)** Flow cytometric analysis of CD36 on CD45neg cells from central and peripheral ccRCC tumor tissue and adjacent kidney. Values were normalized to kidney controls. Representative histogram with geometric mean fluorescence intensities on the right. One-way ANOVA with Dunnett post-test comparing to kidney. *p<0.05. **(F)** Lipidomic analysis of ccRCC tumors and adjacent kidney tissues depicting the percentage of cholesterol esters (CE), triglycerides (TG) and free cholesterol (FC) from all lipids. *p<0.05, Student´s t-test. **(G)** Data from **(F)**, with additional species: lysophosphatidylcholine (LPC), lysophosphatidylethanolamine (LPE), sphingosine based ceramides (Cer), phosphatidic acid (PA), hexosylceramides (Hex-Cer), phosphatidylethanolamine (PE), phosphatidylserine (PS), phosphatidylglycerol (PG), PE based plasmalogen (PE P), phosphatidylinositol (PI), cardiolipin (CL), phosphatidylcholine-ether (PC O), phosphatidylcholines (PC), sphingomyelins (SM), diacylglycerol (DAG). Each column represents one individual ccRCC patient. Color represents signal normalized to row mean. Correlation of CD36 staining data [as in **(B)**] with the TG levels. Pearson´s correlation, two-tailed p-value.

We studied samples from ccRCC patients using conventional immunohistochemistry, multipleximmunofluorescence imaging (MIFI), flow cytometry (FC) and publicly available ccRCC transcriptome data (**Supplementary Figure S1A**). Staining with Oil Red O showed an increased accumulation of lipids in the ccRCC tumor tissue, as compared to healthy kidney. The results of the automated approach generating the area staining intensity were confirmed through observer-based histological score (**Figure 1A**). Next, the expression of CD36, a key lipid-transporting molecule, was assessed. CD36 was increased in ccRCC tumor tissue (**Figure 1B**) and microscopy-based data were validated with expression analyses using RNA sequencing data from the TCGA database (**Figure 1C**). The CD36 expression did not correlate with Oil Red O signals (**Supplementary Figure S1B**). MIFI analyses confirmed CD36 expression in ccRCC tissues (**Figure 1D**; **Supplementary Figure S1C**). Next, we studied freshly resected samples from ccRCC tumor periphery, tumor center andadjacent kidney using FC (**Supplementary Figure S1D**). As opposed to fixed tissues assessed by immunohistochemistry, no robust FC marker for ccRCC cancer cells has been widely established so far, therefore we analyzed the CD36 expression on CD45-negative (CD45neg) cells as a proxy for cancer cells (40, 41). Compared to CD45neg cells from adjacent kidney, tumor samples showed increased expression of CD36 (**Figure 1E**). Lastly, ccRCC tumors accumulated triglycerides (TG) and, by a trend, cholesterol esters (CE). The percentage of free cholesterol (FC) from all lipids in the tumor was decreased by a trend (**Figure 1F**). CD36 expression correlated to intratumoral triacylglycerol (TG) levels by a trend (**Figure 1G**; **Supplementary Figure S1E**).

### ccRCC tumor associated macrophages express CD163

**Figure 2:**
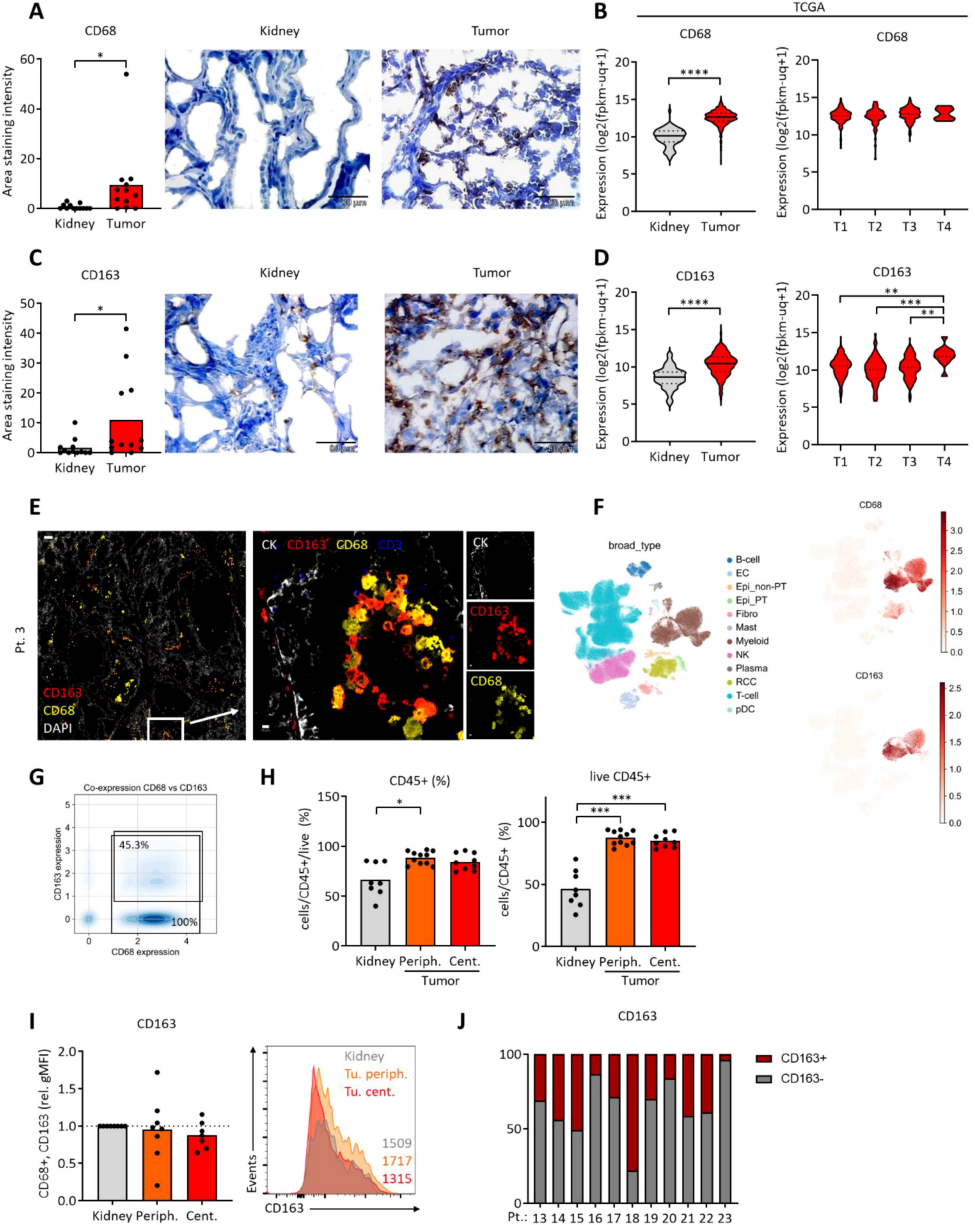
Expression of CD68 and CD163 in ccRCC samples. **(A)** CD68 was assessed using immunohistochemistry. **(B)** Expression of CD68 was assessed using the available TCGA data (KIRC dataset). Tumors were grouped by the pathological tumor size (pT). **(C)** CD163 was assessed using immunohistochemistry. **(D)** Expression of CD163 was assessed using the available TCGA data (KIRC dataset). Tumors were grouped by the pathological tumor size (pT). Student’s t-test for two groups and one-way ANOVA for four groups; *p<0.05, **p<0.01, ***p<0.001,****p<0.0001. **(E)** Multiplex immunofluorescence imaging of ccRCC tumor tissues (representative of three patients) depicting CK (cytokeratin), CD163 and CD68 expression. White bar represents 200 μm (left) and 10 μm (right). **(F)** UMAP depicting clusters of single-cell data showing the expression of CD68 and CD163. Cell type annotations were adopted from the original publication (34). **(G)** Fraction of CD68+ cells co-expressing CD163 in RCC tumor tissue macrophage population. Positivity in scRNA-seq for CD68 and CD163 was defined from raw UMI counts as ≥1 UMI per gene **(F)**. **(H)** Flow cytometric analysis of CD45 on cells from central and peripheral ccRCC tumor tissue and adjacent kidney. **(I)** As in **(H)**, analysis of CD163 on CD68+ cells from central and peripheral ccRCC tumor tissue and adjacent kidney. Values were normalized to kidney controls. Representative histogram with geometric mean fluorescence intensities on the right. One-way ANOVA with Dunnett post-test comparing to kidney. *p<0.05, **p<0.01, ***p<0.001. **(J)** Data from **(I)**, tumor periphery, plotted as individual patients, depicting the portion of CD163+ and CD163neg cells.

Accumulation of lipids and increased expression of CD36 might associate with intratumoral immune cells such as macrophages, which show high lipid metabolic activity and which also react to lipid metabolic changes in tissues. The expression of the macrophage marker CD68 was increased in ccRCC tumor tissues (**Figure 2A**; **Supplementary Figure S2A**). Increased CD68 levels in tumors were validated on the transcriptional level, but CD68 expression was not higher in larger tumors (**Figure 2B**). CD163 which associates with pro-tumorigenic macrophage populations (22) was increased in the ccRCC tumors (**Figures 2C, D**; **Supplementary Figure S2B**). Interestingly, CD163 expression levels increased with tumor size, suggesting its role in tumor promotion (**Figure 2D**).

MIFI analyses revealed a co-expression of CD68 and CD163 in ccRCC tumor tissues (**Figure 2E**; **Supplementary Figures S2D, E**). Next, single cell RNA-sequencing (scRNAseq) data by Li et al. (34) were re-analyzed and confirmed the co-expression of CD68 and CD163 in tumor cells in the myeloid cell cluster (**Figure 2F**). This data set includes non-malignant tissues from ccRCC patients (**Supplementary Figure S2F**), however, cells from adjacent kidney were almost not present in the myeloid cell cluster(**Supplementary Figure S2G**). On average, 45.3% of CD68+ cells also co-expressed CD163 (**Figure 2G**). The expression of TREM2, APOE, C1QA and to a lesser extent MRC1 suggested apro-tumorigenic macrophage phenotype (42, 43) (**Supplementary Figure S2G**).

Next, we studied ccRCC samples using FC. While the infiltration with CD45+ cells (which include CD68+ macrophages) and the proportion of living CD45+ cells was lower in adjacent kidney (**Figure 2H**), the CD163 expression on CD68+ cells was not different and showed a strong variation among the individual patients (**Figures 2I, J**). These data suggest, that CD163+ macrophages are present both in kidney tissues and ccRCC tumors. However, increased numbers of CD45+ cells result in higher absolute numbers of CD163+ macrophages in ccRCC tumors.

Lastly, CCR2 which can mark “M2-like” macrophages positively correlated with CD163and CD14 on ccRCC TAM in FC data (**Supplementary Figure S2H**) and in transcriptomics (**Supplementary Figure S2I**). CCR2 ligands CCL7, CCL8 and CCL13 are negatively prognostic and CCL13 correlates with CD163 in the KIRC TCGA cohort (**Supplementary Figure S2J**).

### High tumoral CD36 expression positively associates with macrophage CD163 levels

**Figure 3:**
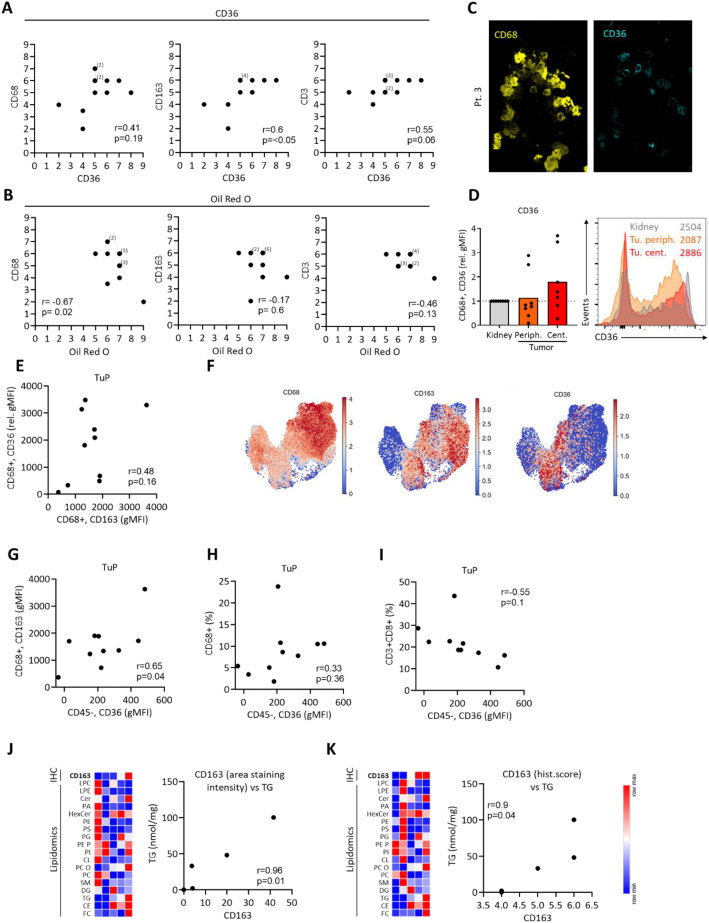
Correlations of CD36 and Oil Red O with immunological markers. Data was obtained as in **Figures 1**, **2**. Observer-based histological scores were used to calculate the correlations. As these scores are ordinal, individual data points may overlap in scatter plots. The number of overlapping values is indicated by numbers in parentheses. Correlation statistics were performed using Pearson´s correlation coefficients, and p-values were two-tailed. **(A)** Correlations of CD36 with CD68, CD163 and CD3. **(B)** Correlations of Oil Red O scores to CD68, CD163 and CD3. **(C)** Multiplex immunofluorescence imaging of ccRCC tumor tissues (representative of three patients) depicting CD68 and CD36 expression. **(D)** Flow cytometric analysis of CD36 on CD68+ cells from central and peripheral ccRCC tumor tissue and adjacent kidney. Values were normalized to kidney controls. Representative histogram with geometric mean fluorescence intensities on the right. One-way ANOVA with Dunnett post-test comparing to kidney, n.s. **(E)** As in **(D)**, correlation of CD36 and CD163 expression on CD68+ cells. **(F)** UMAP depicting macrophage cluster with expression of CD68, CD163 and CD36 (34). **(G-I)** Flow cytometric analysis of peripheral tumor tissues, correlating the expression of CD36 on CD45neg cells to **(G)** CD163 on CD68+ cells, **(H)** the CD68+ cell frequencies and **(I)** the frequencies of CD3+ CD8+ cells. Pearson´s correlation, two-tailed p-value. **(J, K)** Lipidomics performed on five ccRCC tumors with correlations of CD163 expression (IHC, area staining intensity in J; histological score in K) and the levels of triacylglycerol (TG).

To study potential links between ccRCC lipid metabolism and its immune infiltrate, we correlated the obtained microscopy data sets. While the CD36 levels did not correlate with CD68 expression, the correlation of CD163 and CD36 was highly significant, suggesting a favoring of pro-tumorigenic macrophage subpopulations in ccRCC tumors with increased CD36 levels (**Figure 3A**). While the infiltration of CD3 T cells was increased in ccRCC tumors, as compared to normal kidney tissues (**Supplementary Figure S2C**), the correlation of CD3 and CD36 was not significant (**Figure 3A**). Oil Red O staining was used in correlations to immune cell markers next. Interestingly, we observed a negative correlation of Oil Red O and CD68. In contrast, no correlation was observed while comparing Oil Red O to CD163 and CD3 expression (**Figure 3B**).

CD36 was expressed on ccRCC tumor cells (**Figure 1**). However, CD36 can also be expressed by macrophages (7, 8). Accordingly, MIFI confirmed CD36 expression on CD68+ cells in ccRCC tissues (**Figure 3C**). Using FC, we assessed the CD36 expression on macrophages, but did not detect a difference in tumor tissues as compared to adjacent kidney (**Figure 3D**). Furthermore, macrophage CD36 expression did not correlate to CD163 (**Figure 3E**) and scRNAseq data showed that only a subpopulation of CD163+ macrophages co-expressed CD36 (**Figure 3F**; **Supplementary Figures S3A, B**). However, we detected a positive correlation of CD36 on CD45neg cells with CD163 on macrophages in ccRCC tumors. Importantly, this was not the case with the overall frequencies of CD68+ cells (**Figures 3G, H**). Similar to CD163, CD36 on CD45neg cells correlated positively with the frequency of CCR2+macrophages and by a trend with their CD14 expression (**Supplementary Figures S3C, D**). In addition, we observed a trend towards a lower infiltration with CD3+CD8+ T cells in tumors with high expression of CD36 in CD45neg cells (**Figure 3I**).

Lastly, lipidomic analyses revealed a positive correlation between the intratumoral triacylglycerol (TG) levels and the CD163 expression (**Figures 3J, K**).

### CD147 is co-expressed with CD36 in ccRCC

**Figure 4:**
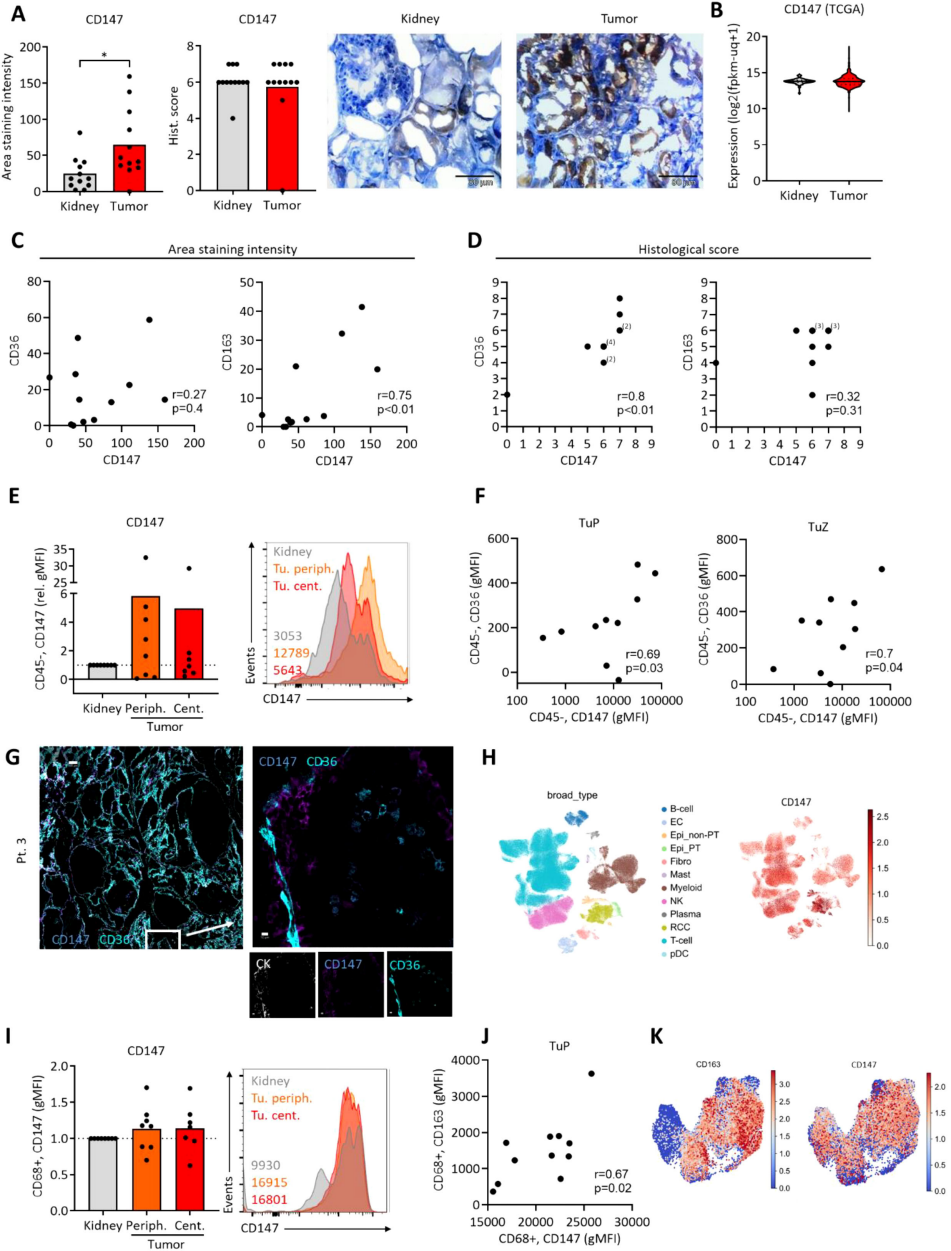
Expression of CD147 in ccRCC samples. **(A)** CD147 was assessed using immunohistochemistry. Student’s t-test, *p<0.05. **(B)** Expression of CD147 was assessed using the available TCGA data (KIRC dataset). **(C)** Correlations of CD147 expression to CD36 and CD163 using area staining intensity. **(D)** Correlations of CD147 expression to CD36 and CD163 using observer-based histological scores. As these scores are ordinal, individual data points may overlap in scatter plots. The number of overlapping values is indicated by numbers in parentheses. Correlation statistics were performed using Pearson´s correlation coefficients, and p-values were two-tailed. **(E)** Flow cytometric analysis of CD147 on CD45neg cells from central and peripheral ccRCC tumor tissue and adjacent kidney. Values were normalized to kidney controls. Representative histogram with geometric mean fluorescence intensities on the right. **(F)** As in **(E)**, correlations of CD147 expression on CD45neg cells to CD36 in tumor periphery and tumor center. **(G)** Multiplex immunofluorescence imaging of ccRCC tumor tissues (representative of three patients) depicting CD36 and CD147 expression. White bar represents 200 µm (left) and 10 µm (right). **(H)** UMAP depicting clusters of single-cell data from ccRCC patients showing the expression of CD147. Cell type annotations were adopted from the original publication (34). **(I)**. Flow cytometric analysis of CD147 on CD68+ cells from central and peripheral ccRCC tumor tissue and adjacent kidney. Values were normalized to kidney controls. Representative histogram with geometric mean fluorescence intensities on the right. **(J)** As in **(I)**, correlation of CD163 and CD147 expression on CD68+ cells. Pearson´s correlation, two-tailed p-value. **(K)** UMAP visualization of CD163 and BSG (CD147) expression on myeloid cells from ccRCC tumors, cell type annotations were adopted from the original publication (18).

Next, the expression of CD147, a transmembrane protein regulating several metabolic transporters was assessed. While its levels were increased in tumor tissues as assessed by the area staining intensity, the observer-based data and transcriptome data did not show a difference (**Figures 4A, B**). Next, we correlated the expression of CD147 to the lipid transporter CD36 and the macrophage marker CD163. While we observed a positive correlation of CD147 and CD163 in the area staining intensity data, this could not be validated in the observer-based histological score. Conversely, CD147 correlated positively with CD36 in the histological score analysis, but not in the area staining intensity data (**Figures 4C, D**). Furthermore, CD147 did not correlate with the Oil Red O intensity (**Supplementary Figure S4A**).

Using FC, we studied the CD147 expression on the single-cell level. While the variation of CD147 expression on CD45neg cells was high, there was a trend toward its increased expression in tumor tissues (**Figure 4E**). Confirming the data from the histological score analysis, we observed a positive correlation of CD147 and CD36 on CD45neg cells from both peripheral and central tumor tissues (**Figure 4F**). As opposed to CD36, the expression of CD147 on CD45neg cells did not correlate with macrophage CD163 expression (**Supplementary Figure S4B**). MIFI confirmed the expression of CD147 and CD36 in ccRCC tissues (**Figure 4G**; **Supplementary Figure S4C**). scRNAseq data showed a high basal expression of CD147 across all cell types, with a strong enrichment in RCC tumor cells (**Figure 4H**).

Lastly, we studied the expression of CD147 on ccRCC macrophages. FC analyses did not detect a different CD147 expression on tumor macrophages, as compared to those from adjacent kidney samples (**Figure 4I**). However, CD147 expression positively correlated with the expression of CD163 in FC (**Figure 4J**) and scRNAseq analyses (**Figure 4K**).

**Figure 5:**
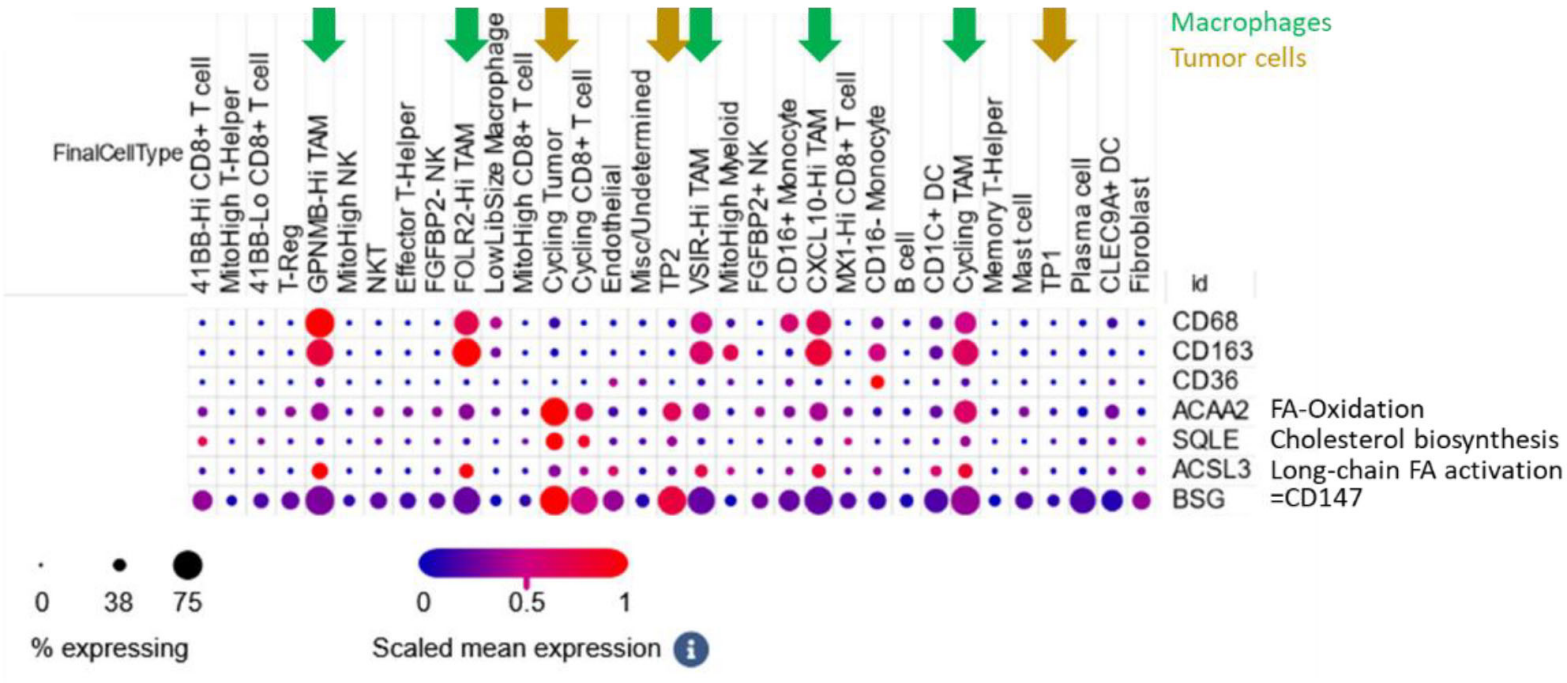
Single cell RNA seq of ccRCC tumors. Data by Bi et al. (18) was visualized and via the Single Cell Portal (39). Cell types, including immune subpopulations, were annotated as defined by the original authors. Expression of metabolic (ACAA2, SQLE, ACSL3, CD36) and immunologic (CD68, CD163, CD147) genes was examined across these distinct immune compartments. For the characterization of the immune cell populations, please refer to Bi et al. (18).

To further analyze the macrophage sub-populations in ccRCC, we studied a second cohort of single cell RNA sequenced ccRCC tumors by Bi et al. (18). Here, single cell RNA sequencing has been performed and individual cells were assigned to several subpopulations. In addition to the macrophage markers CD68 and CD163, we assessed the expression of CD147, CD36, ACAA2 (a prominent gene involved in fatty acid oxidation), SQLE (a major representative of cholesterol biosynthesis), ACSL3 (key regulator of long-chain fatty acid activation). All macrophage sub-populations showed prominent CD163 expression, suggesting their pro-tumorigenic phenotype. Furthermore, we observed a strong expression of CD147 in tumor cells and in macrophages (**Figure 5**). ACSL3 was mainly expressed in macrophages and SQLE in tumor cells, suggesting a compartment-specific lipid metabolism. ACAA2 was expressed by both tumor cells and macrophages, suggesting a shared fatty acid oxidation pathway.

## Discussion

Clear cell renal cell carcinoma (ccRCC) is a metabolically distinct and immunologically active tumor type, yet current treatments—including immune checkpoint inhibitors—often fail to induce durable responses in many patients. This highlights a pressing clinical need to better understand the tumor microenvironment (TME) and to identify novel mechanisms of immune evasion and resistance. Emerging evidence suggests that tumor metabolism and immune regulation are closely linked, particularly through altered lipid handling in both malignant and non-malignant cells. In this study, we analyzed the lipid metabolic regulators CD36 and CD147 with a focus on tumor-associated macrophages (TAMs).

We observed a prominent lipid accumulation together with increased CD36 expression in ccRCC at both the protein and transcript level, in agreement with previous reports (9). Interestingly, CD36 expression does not correlate with Oil Red O staining in our analyses, suggesting that fatty acid uptake and neutral lipid storage represent distinct metabolic states. CD36 likely marks a dynamic, oxidative phenotype characterized by active fatty acid import and utilization rather than passive lipid deposition. This interpretation aligns with the metabolic profile of VHL-deficient, HIF-driven tumors that rely on fatty acid oxidation for energy production and redox control.

CD163+ tumor-associated macrophages (TAMs) were abundant in ccRCC. Although the relative proportion of CD163+ macrophages from all CD68+ cells does not differ markedly from adjacent kidney in flow cytometric analyses, the overall leukocyte infiltration is higher in tumors, leading to greater absolute numbers of CD163+ cells. These TAMs express additional immunosuppressive markers, consistent with an M2-like, tumor-promoting phenotype within the lipid-rich microenvironment. Specifically, single cell RNA sequencing data showed a prominent expression of TREM2, APOE, C1QA, and to a lesser extent MRC1, all markers associated with an M2-like phenotype (42, 43).

Nevertheless, the interpretation of tumor versus “normal” kidney tissue must be approached with caution. The so-called normal samples in nephrectomy specimens are obtained from regions adjacent to the tumor and therefore do not represent truly healthy renal parenchyma. These areas can be affected by altered perfusion, local inflammation, and systemic metabolic stress associated with the tumor-bearing kidney (44–46). Moreover, renal function frequently declines in the presence of ccRCC, leading to ischemia and metabolic remodeling even in macroscopically uninvolved tissue. Consequently, differences between tumor and “normal” kidney should be interpreted as relative rather than absolute, reflecting a gradient of metabolic and immune changes rather than a strict dichotomy.

A positive correlation between CD36 expression on CD45neg cells and CD163 expression on TAMs suggests that tumor lipid metabolism favors the differentiation or persistence of M2-like macrophages. In addition, tumors with high CD36 expression tend to contain fewer CD8 T cells, indicating that lipid metabolic reprogramming may contribute to a broader immune-suppressive milieu characterized by reduced cytotoxic infiltration. In flow cytometry, we used the CD45neg population as a surrogate for tumor cells because no single robust surface marker reliably distinguishes ccRCC cells from normal renal epithelium in fresh tissue in flow cytometry as opposed to fixed tissues assessed by immunohistochemistry. Cytokeratin and CA9 can indicate epithelial or RCC identity but are not consistently preserved under enzymatic digestion. Therefore, exclusion of CD45+ immune cells provided the most consistent strategy to define the tumor-cell compartment for comparative analyses.

CD36, but not Oil Red O staining, correlated with CD163 intensity on macrophages, suggesting that macrophage polarization is linked to tumor lipid metabolism rather than overall lipid accumulation. Why could an increased CD36 expression in tumors favor M2-like macrophages? Tumor cells with high CD36 may compete for extracellular fatty acids, creating a metabolically altered microenvironment characterized by hypoxia and nutrient imbalance. Such conditions could favor oxidative, M2-like macrophage states, even in the absence of excess lipid storage, but these hypotheses will be tested in future studies.

Nevertheless, CD36 was also expressed on TAMs and positively correlated with TAM expression of CD163. These data suggest that lipid handling is linked to macrophage polarization within ccRCC. CD36 expression on TAMs might be driven by lipid-related cues in the tumor microenvironment. Yang et al. showed that lipid-enriched vesicles in tumors are preferentially taken up into macrophages via CD36, that fuel macrophages and trigger their tumor-promoting activities (47). Another study showed that TAMs express CD36 and use fatty acid oxidation, leading to production of reactive oxygen species and to transcription of genes mediating a pro-tumor function (M2-like phenotype) (7). Thus, we speculate, that CD36 upregulation in ccRCC TAMs may reflect their adaptation to lipid overload and engagement in clearance and tissue-remodeling functions within ccRCC.

CD147 has been associated with tumor progression and immune evasion as well as immune dysfunction (12, 13). Furthermore, CD147 is a regulator of fatty acid metabolism (14) and lipid trafficking (15). We and others showed that CD147 expression can be induced through hypoxia (12). In addition, metabolic factors, such as low-density lipoprotein can induce CD147 (48). CD147 was also elevated in ccRCC and partially overlapped with CD36 and CD163 depending on the cellular context. Our analyses were limited by different results obtained when using the subjective scoring system and the objective area staining intensity. Nevertheless, correlations between CD147 and CD36 are most evident in CD45neg cells, whereas CD147 and CD163 associate within macrophages. These patterns point to a broader lipid metabolism–linked network involving both CD36 and CD147 that may support immune evasion. In VHL-mutant, HIF-active tumors, this metabolic state is likely to promote an environment permissive for M2-like macrophage polarization. Lastly, single-cell RNA sequencing data, published by Bi et al. (18) confirmed CD147 expression on tumor cells and macrophage subsets and suggest a compartment-specific expression of key lipid metabolism genes—ACSL3 (fatty acid activation) in macrophages, SQLE (cholesterol biosynthesis) in tumor cells, and shared ACAA2 (fatty acid oxidation) expression in both. These findings point to a complex interaction between tumor lipid metabolism and the immune microenvironment.

Together, the data support a model in which tumor-intrinsic lipid metabolism contributes to immune suppression in ccRCC. While the mechanisms remain to be defined, the consistent association between lipid metabolic markers and macrophage polarization highlights a metabolic–immune axis that could be therapeutically targeted.

Several limitations should be noted. The functional role of circulating CD163+ macrophages was not addressed, although single-cell RNA sequencing indicates their presence in peripheral blood. Moreover, discrepancies between transcript and protein levels—such for CD36, which can be affected by microRNA and long non-coding RNAs (49) —suggest post-transcriptional regulation and underscore the need for integrated multi-omic analyses to resolve metabolic–immune interactions in ccRCC. Lastly, low number of included patients hinders a generalization of our observations to the broad patient collective.

Therapeutically, targeting CD36 or CD147 could disrupt this tumor-promoting circuit. CD36 blockade—via antibodies or small-molecule inhibitors—may impair lipid uptake, suppress tumor proliferation, and prevent the immunosuppressive polarization of TAMs. Similarly, inhibiting CD147 could interfere with tumor metabolic flexibility and alter macrophage function. Preclinical studies in other cancer types have shown that CD36 inhibition can reduce metastasis and improve immunotherapy responses (5). However, given the physiological roles of these proteins in normal tissues, selective targeting strategies, such as bi-specific antibodies, will be necessary to avoid off-target effects. Combining CD36 or CD147 inhibitors with immune checkpoint blockade may represent a promising therapeutic strategy, particularly in patients with lipid-rich, CD36-high/CD163-high tumors.

One strength of our study is the integration of multiple analytical approaches, combining histological staining, transcriptomics, flow cytometry and single-cell RNA sequencing. The limitations of our study include its correlative nature. Furthermore, functional experiments will be required in future studies to determine whether CD36 and CD147 actively drive macrophage polarization or are markers of the lipid-rich microenvironment. Additionally, discrepancies between quantification methods (e.g., staining intensity vs. observer-based scoring) underscore the need for standardized, reproducible tools to evaluate protein expression in tissue samples.

Future studies should focus on mechanistic investigations to define the causal role of CD36 and CD147 in TAM differentiation and function. Furthermore, analyzing patient samples before and after immunotherapy could determine whether high CD36/CD163 expression correlates with resistance, identifying candidates who may benefit from metabolic targeting. Finally, longitudinal studies are needed to explore whether the lipid-immune landscape evolves during disease progression or treatment, which may inform optimal timing for therapeutic intervention.

In summary, our study reveals that lipid accumulation and upregulation of CD36 and CD147 are features of the ccRCC microenvironment and co-occur with the presence of pro-tumorigenic, CD163+ macrophages. While we cannot assign a direct tumor-specific or a macrophage-specific role of CD36 and CD147 for ccRCC progression, our findings suggest a potential metabolic-immune axis that could be therapeutically targeted to reprogram TAMs and disrupt tumor-supportive conditions. By identifying CD36 and CD147 as candidate regulators of immunosuppressive ccRCC microenvironment, we provide a rationale for future studies investigating metabolic inhibitors as adjuncts to immunotherapy in ccRCC. Elucidating the precise molecular mechanisms of this crosstalk and validating these targets in functional models will be critical next steps toward translating these insights into clinical strategies.

## Data Availability

This study used publicly available datasets, which are cited in the Methods section. Further inquiries regarding the original data generated in this study should be directed to the corresponding author.
