# Supplementary material for: Increased expression of CD36 and CD163 in clear cell renal cell carcinoma suggests an association between lipid transport and an “M2-like” macrophage phenotype
Source: Front Immunol. 2026 Mar 2;17:1773666. doi: 10.3389/fimmu.2026.1773666 (PMC12989337; doi:10.3389/fimmu.2026.1773666)
Supplement: Supplementary file 1 [file Presentation1.pdf]

Supplementary Data

Fig. S1

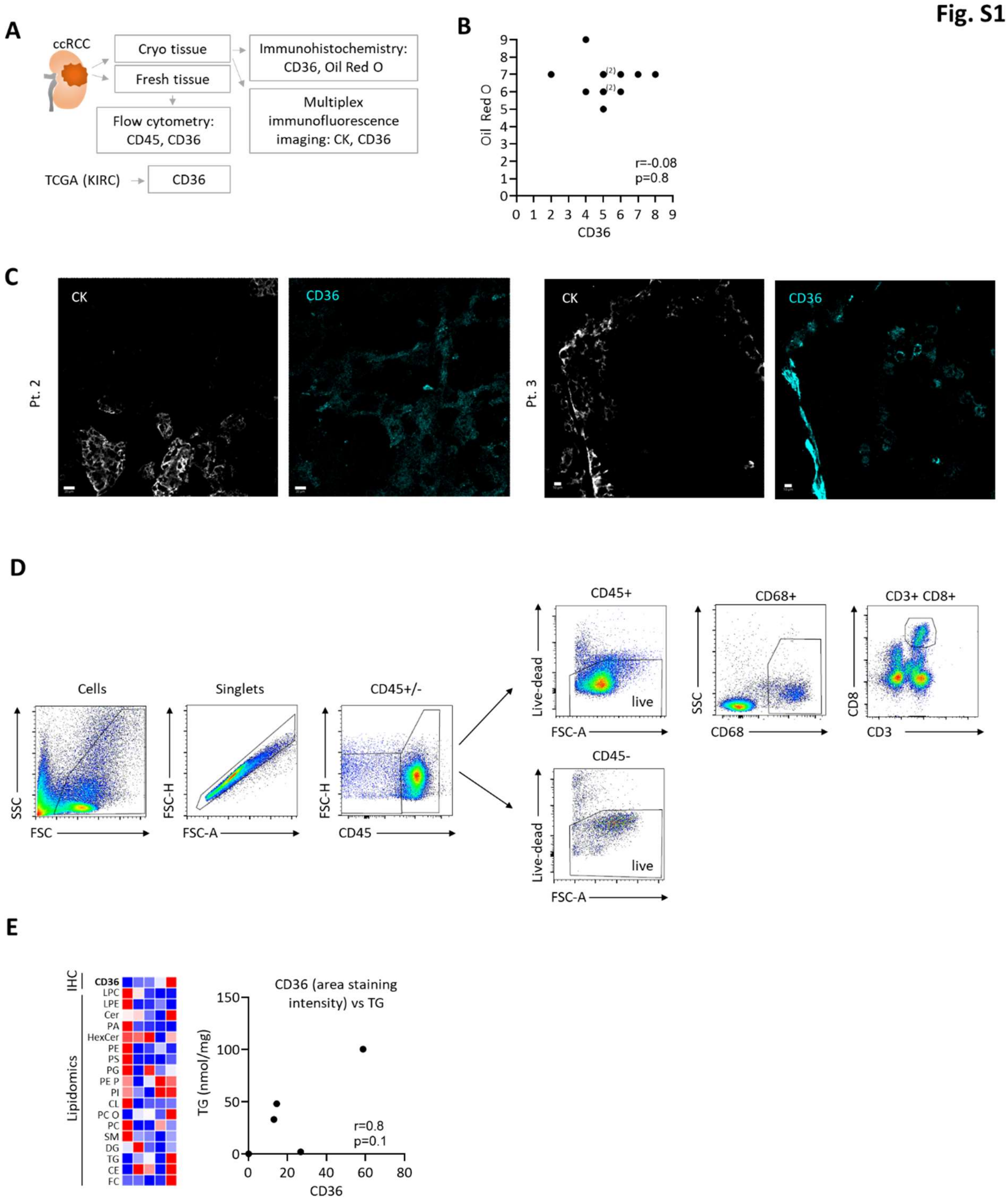

Figure S1. **CD36 Expression in ccRCC.** (**A**) Schematic. (**B**) Immunohistochemistry (observer score) assessing Oil Red O and CD36 in ccRCC tumor samples. Pearson´s correlation, two-tailed p-value. (**C**) Multiplex immunofluorescence imaging of ccRCC tumor tissues depicting CK (cytokeratin) and CD36 expression. (**D**) Flow cytometric analysis of ccRCC tissues, gating example. (**E**) Lipidomics performed on five ccRCC tumors

with correlations of CD36 expression (IHC, area staining intensity and the levels of triacylglycerol (TG). For the list of lipid species, refer to Fig. 1G.

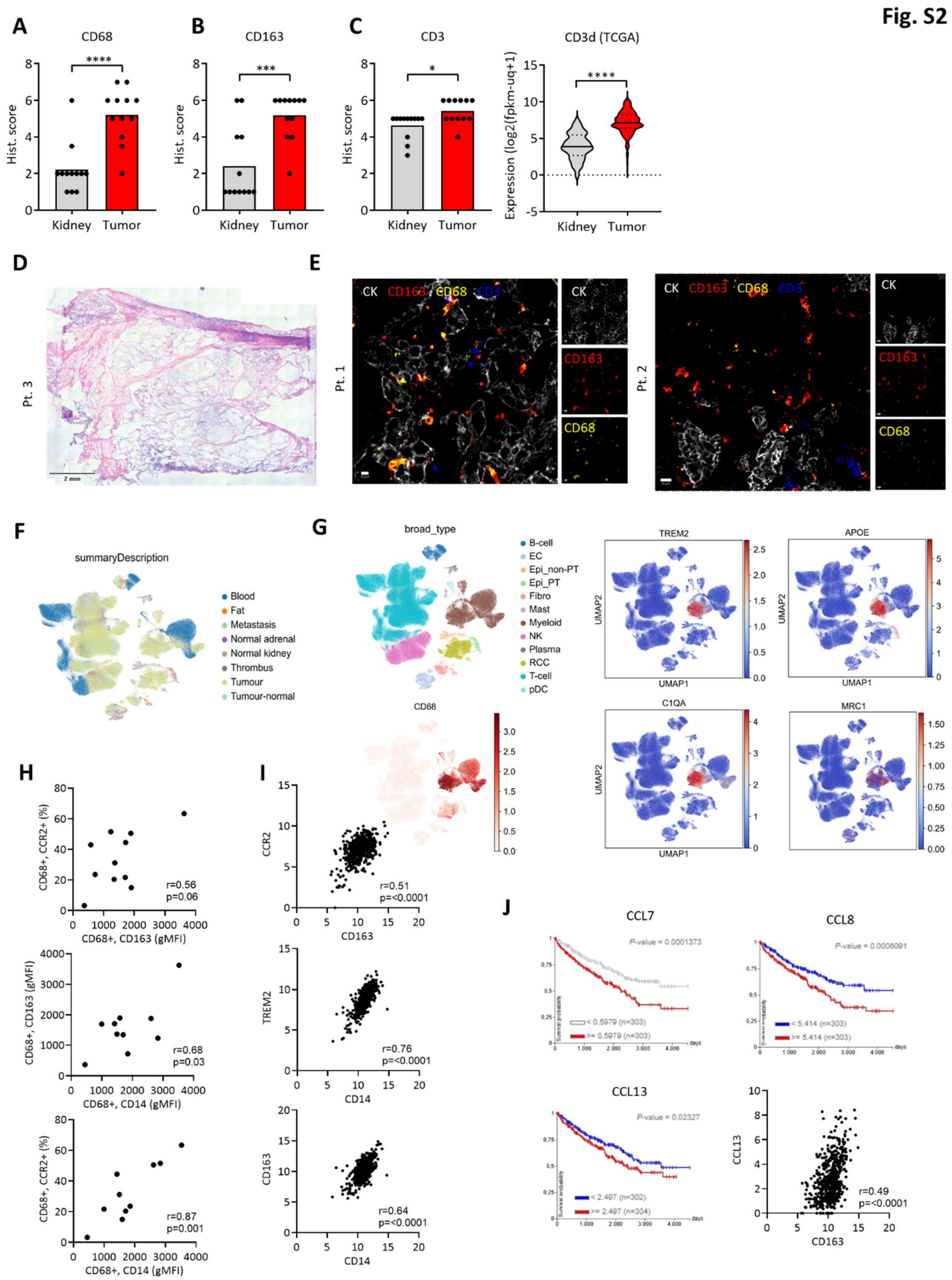

Figure S2. **Expression of immune cell markers in ccRCC samples**. (**A**) As in Fig.2A, but using the observer-based histological score. (**B**) As in Fig.2C, but using the observer-based histological score. (**C**) CD3 was assessed using immunohistochemistry. CD3d, as a representative sub-unit of CD3 was assessed using the

available TCGA data (KIRC dataset). * $p<0.05$, *** $p<0.001$,**** $p<0.0001$. (**D**) Hematoxylin and Eosin staining of Pt. 3 (please refer to Fig. 2E). (**E**) Multiplex immunofluorescence imaging of ccRCC tumor tissues depicting CK (cytokeratin), CD163, CD68 and CD3 expression. (**F**) UMAP depicting origin of cells including in scRNAseq analyses. (**G**) As in (F), UMAP depicting clusters of single-cell data showing the expression of CD68, TREM2, APOE, C1QA and MRC1. Cell type annotations were adopted from the original publication [34]. (**H**) Correlations of flow cytometric data assessing the expression of CCR2, CD163 and CD14 on CD68+ ccRCC cells. (**I**) TCGA data from the KIRC cohort correlating the expression of TREM2, CD14, CD163 and CCR2. (**J**) TCGA data showing the survival of ccRCC patients based on the expression of CCL7, CCL8, CCL13 and showing a correlation of CCL13 and CD163 expression.

Fig. S3

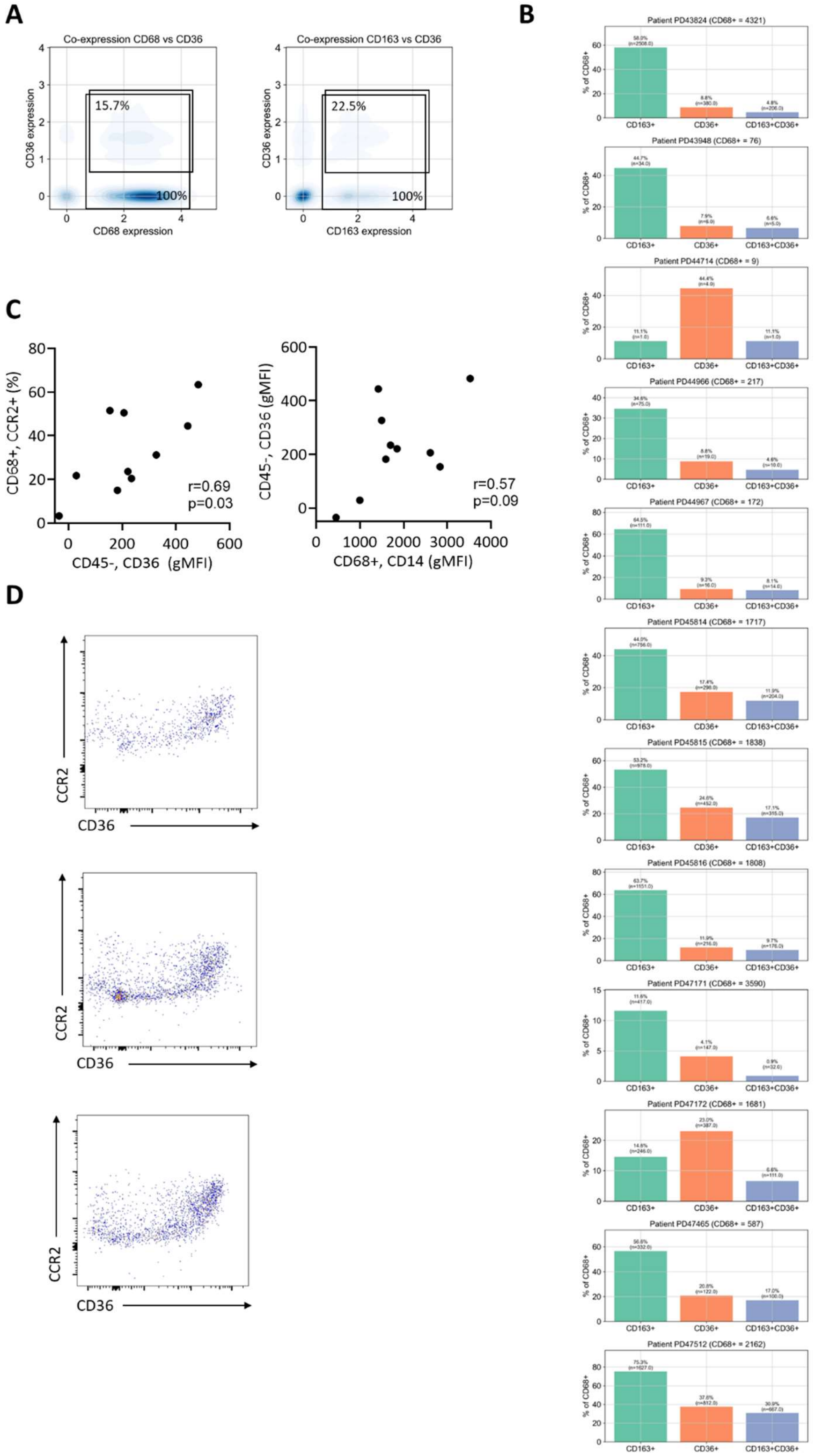

Figure S3. Expression of CD68, CD163 and CD36 in the myeloid cell cluster on average (**A**) and in individual ccRCC patients (**B**). Data and cell type annotations were adopted from the original publication [34]. Values in (B) represent the percentage of all myeloid cells being defined as CD36+, CD163+ or CD36+163+. (**C**)

Correlations of flow cytometric data assessing the expression of CCR2 and CD14 on CD68+ cells with the expression of CD36 on CD45-negative cells in ccRCC tumors. (**D**) Samples as in (C), comparative depiction of CD36 and CCR2 on CD68+ cells from ccRCC tumors. Representative data from three patients.

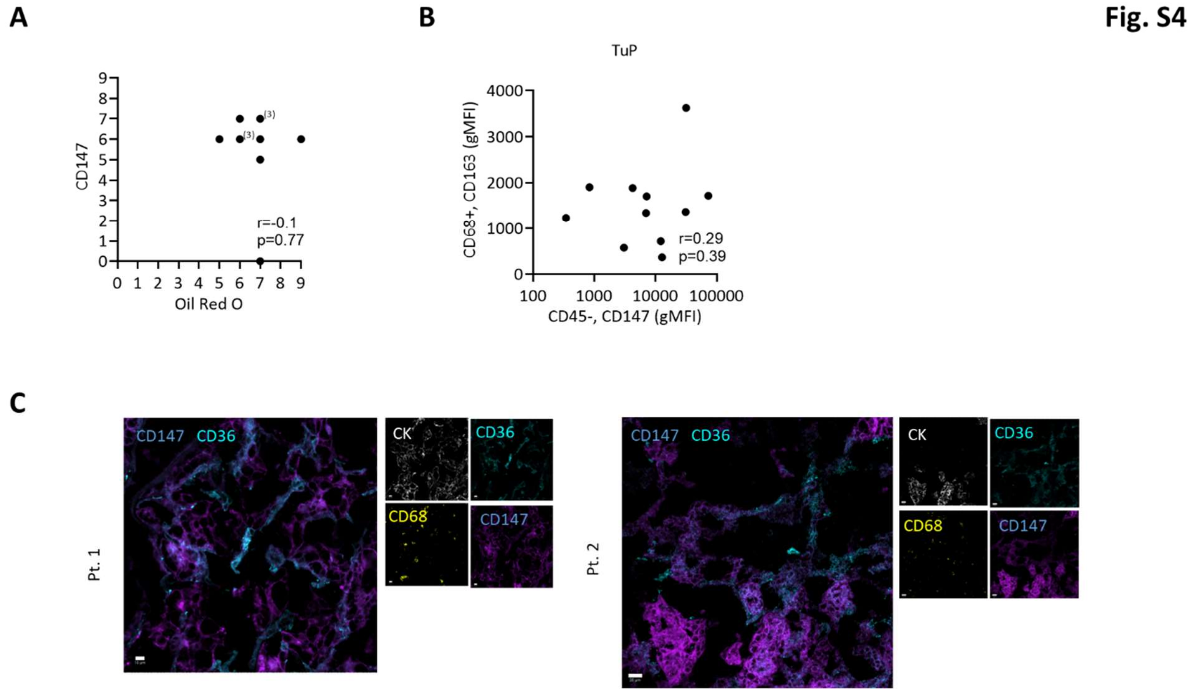

Figure S4. (**A**) CD147 expression and Oil Red O intensity was assessed using immunohistochemistry. (**B**) Flow cytometric analysis of CD147 on CD45neg cells and CD163 on CD68+ cells from peripheral ccRCC tumor tissue. Pearson´s correlation, two-tailed p-value. (**C**) Multiplex immunofluorescence imaging of ccRCC tumor tissues depicting CD147, CD36, CK (cytokeratin) and CD68 expression.

Suppl. Table 1

**Age**	69.3±11.9
**Gender**	
Male	56%
Female	44%
**Stage**	
pT1a-pT2	31%
pT3a-pT4	69%
**Grade**	
G1	13%
G2	63%
G3	13%
**N-Status**	
N0	44%
N1	6%
N2	6%
ND	44%
**% malignant tissue** of tumor sample	74% ± 23%
Clinical information except histology not available	17%

Supplemental Table 1. Clinical characteristics of ccRCC patients. Grading was performed by an experienced pathologist. +/- values represent standard deviation.
